# Recognition and reporting of neglected tropical diseases by primary health care workers in Ibadan, Nigeria

**DOI:** 10.11604/pamj.2021.38.224.20576

**Published:** 2021-02-26

**Authors:** Daniel Chukwuyere Emeto, Adetokunbo Taophic Salawu, Mobolaji Modinat Salawu, Olufunmilayo Ibitola Fawole

**Affiliations:** 1 Department of Epidemiology and Medical Statistics, Faculty of Public Health, College of Medicine, University of Ibadan, Ibadan, Nigeria

**Keywords:** Neglected tropical diseases, knowledge, recognition, reporting, primary healthcare workers, schistosomiasis, onchocerciasis

## Abstract

**Introduction:**

neglected tropical diseases (NTDs) are serious public health problem worldwide. Primary healthcare (PHC) workers are important in the prevention and control of these diseases. Accurate diagnosis and timely reporting are essential for effective public health response. The study assessed the knowledge of PHC workers on NTDs and identified factors influencing its recognition and reporting.

**Methods:**

the study was a hospital based cross-sectional survey. A multistage sampling technique was used to select 327 healthcare workers from sixty-six PHC facilities in Ibadan, Oyo State, Nigeria. A semi-structured, self-administered questionnaire was used to elicit information on respondent´s socio-demographic characteristics, knowledge, recognition and reporting of NTDs. Data analysis were done using independent sample t-test, analysis of variance and logistic regression with p-value set at 0.05.

**Results:**

one hundred and eighty seven (57.2%) respondents had good knowledge of NTDs. The NTDs most known were; Schistosomiasis (78%), Rabies (64.5%), and Onchocerciasis (57.2%). Urban healthcare workers had higher knowledge score on NTDs (p= 0.018). Young age (AOR= 3.40, CI= 1.20 -9.63), length of practice (AOR=4.65, CI= 1.20-18.09) and previous training on NTDs (AOR = 7.09, CI= 3.15 - 15.93) significantly predicted NTDs recognition, while availability of reporting forms (AOR= 3.17, CI=1.07 - 9.35), training on disease reporting (AOR= 3.41, CI= 11.17 - 9.92) and feedback on previous diseases reported (AOR= 4.12, CI= 1.32-12.80) were significant predictors of reporting NTDs.

**Conclusion:**

the healthcare workers knowledge on NTDs was inadequate. Periodic training and continued education on NTDs are recommended. PHC workers also require supportive supervision.

## Introduction

Neglected tropical diseases are heterogeneous groups of tropical infections common in low income countries where access to clean water, sanitation, good housing, health care, and vector control are limited. They are caused by helminth, bacteria, virus, and protozoa and are characterized by chronic, disfiguring and disabling conditions [1]. Despite their serious health consequences, they are overshadowed by HIV/AIDS, tuberculosis, and malaria which receive greater attention for treatment, research and funding programs [2]. There has been differences in the diseases classified as NTDs by the World Health Organization (WHO), the Center for Disease Control and prevention (CDC), and infectious disease experts. The WHO recognizes twenty NTD diseases, while the CDC classified 18 diseases as such [3,4]. The Nigeria Federal Ministry of Health (FMOH) recognizes nine diseases namely; Trachoma, Buruli ulcer, Trypanosomiasis, Dengue Fever, Schistosomiasis, River Blindness, Lymphatic Filariasis, Onchocerciasis and Soil Transmitted Helminthes [5]. Globally, NTDs are found in 149 countries and are second to HIV/AIDS in terms of number of people affected by the diseases. There are over one billion people with at least an NTD (including more than 500 million children) and 500, 000 deaths yearly. Yet, NTDs receives only 0.6% health fund of official development assistance unlike HIV/AIDS, malaria and tuberculosis that collectively receive above 50% [1, 6]. NTDs are among the top 10 leading causes of years of healthy life lost to long-term disability and premature deaths worldwide [1,7].

In sub-Saharan Africa, more than 500 million people are affected by soil-transmitted helminth, Schistosomiasis, lymphatic filariasis, trachoma and onchocerciasis [7,8]. Hookworm affects 40 to 50 million school-aged children and about seven million pregnant women [9,10]. Despite the remarkable progress in the effort against NTDs, these diseases still persist among people in many low income countries. Ignorance and lack of the skill to identify and treat NTDs have been one of the many reasons that have allowed these infectious diseases to flourish especially in rural communities [11]. Additionally, the disease burden has proved difficult to quantify due to under-reporting, focal clustering and poly-parasitism [12,13]. Worldwide, the highest burden of cases of onchocerciasis and schistosomiasis are seen in Nigeria. Both intestinal and urogenital schistosomiasis are endemic in the country [14,15]. Nigeria also has the greatest number of cases of lymphatic filariasis in Africa and has 18 million people at risk of trachoma, with almost 1.3 million people infected with trichiasis [16]. Hence, it is necessary to eliminate NTDs to achieve the Sustainable Development Goals in Nigeria [17]. Primary healthcare workers play an important role in the detection and reporting of NTDs because they are often the first contact between the community members and health facilities. Their engagement and accurate knowledge is essential for effective diagnosis, control and other interventions targeting NTDs. Thus, the study assessed the knowledge of primary health care workers on NTDs and identified the factors influencing its recognition and reporting in Oyo State, Nigeria.

## Methods

**Study area**: this study was conducted in Ibadan, Oyo State, Nigeria. Ibadan is the most populous city in the State with a population of over 3 million people as at 2011. It is the third most populous city in Nigeria and the country's largest city based on geographical area [18]. Ibadan metropolis has 273 public health facilities which comprises of primary health care facilities, secondary and tertiary health facilities [19]. Ibadan metropolis has 11 local government areas (LGAs) comprising of 5 urban and 6 semi-urban LGAs [18].

**Study design**: a hospital based cross-sectional study design was employed for the survey.

**Study population**: the study population were health workers including doctors, nurses, community health extension workers, community health officers, and medical record officers´ of public PHCs in Ibadan. Healthcare workers who had less than 6 months´ work experience were excluded from the study because they have insufficient information on the channels of reporting diseases.

**Sample size determination**: the sample size was estimated using Leslie Kish formula for cross-sectional survey with a single proportion. Assuming a 95% level of confidence, 5% precision, and 75.0% proportion of recognition of cutaneous leishmaniasis among physicians in healthcare facilities in Sokoto, Nigeria [20]. After adjusting for a 10% non-response rate, the sample size was 320 primary health workers.

**Sampling technique**: a multistage sampling technique was used to select respondents for the study. Stage one involved selecting 6 LGAs (3 urban and 3 semi-urban) from the 5 urban and 6 semi-urban LGAs in Ibadan metropolis using simple random sampling. Stage two involved selecting 11 PHC facilities from each of the selected LGAs using all PHC facilities in each of the selected LGA as the sampling frame. The selection was done by simple random sampling. Stage three involved selecting 5 eligible health workers from each selected facilities for the study by simple random sampling.

**Study instrument**: a semi-structured, self-administered, fifty-five item questionnaire was used to elicit information from the respondents. The reliability of the instrument was determined by pre-testing the research instrument among 33 PHC workers at Oyo East LGA of the State in September 2018. Oyo East is a different location from the study area although shares similar characteristics with primary healthcare workers in Ibadan. Following the pretest the questionnaire was divided into four (4) sections namely: Respondent socio-demographic characteristics including age, sex, marital status; Knowledge on NTDs; Factors influencing the recognition of NTDs and Factors influencing the reporting NTDs.

**Data collection**: a total of 327 primary health care workers participated in the study. The data collection was carried out between November and December 2018. The data was collected at the health workers respective health facilities during their working hours.

**Study variables**: the dependent variables were knowledge of NTDs, recognition of NTDs, and reporting of NTDs. The independent variables included socio-demographic variables such as age (<20, 20-29, 30-39, 40-49 and ≥50 years), sex (male/female), work experience (<5, 5-9, 10-14, 15-19, and ≥20 years), training (yes/no), availability of reporting forms (yes/no), tools for NTDs diagnosis (yes/no), incentives (yes/no), feedbacks (yes/no), and supervisions (yes/no).

**Data management**: the data was entered and analyzed using IBM Statistical Package for Social Sciences software version 21.0. Demographic variables such as age, sex, religion, and education were summarized using descriptive statistics. Continuous variables were summarized as mean and standard deviation, while categorical variables were summarized using frequencies and proportions. To assess the knowledge on NTDs, 29 questions with yes, no and I don´t know responses were included in the questionnaire. Each correct response was awarded a mark, while incorrect responses or those who answered I don´t know were scored 0 mark, thereby resulting in a range of 0 to 29 possible scores. The mean score was used to dichotomize the knowledge score into good and poor knowledge. Respondents whose scores were 16 and above were categorized as having good knowledge, while scores below 16 were regarded as poor knowledge of NTDs. The independent sample t-test, analysis of variance and Tukey´s Honestly Significant Difference (HSD) post hoc test were used to compare the differences in mean of the knowledge scores on NTDs by respondent´s socio-demographic variables. NTDs recognition was measured by asking respondent if they can identify these diseases when a patient present with one, this was recorded as a yes or no response. Reporting of NTDs was measured by asking the respondents if they had reported identified NTDs to the disease surveillance and notification officer (DSNO). Logistic regression analysis was used to determine the variables that predict recognizing and those that predict reporting of NTDs by the study respondents. To control for confounding variables, multivariate logistic regression analysis was done.

**Ethical considerations**: ethical approval was obtained from the Oyo State Ministry of Health Research Ethical Review Committee (Ref. No. AD 13/479/1052). Permission was obtained from the Medical Office of Health (MOH) of each of the participating LGA. Participation was voluntary and a written informed consent was obtained from the respondents after adequate explanation of the study procedure. All personal identifiers were removed from the questionnaire and confidentiality was ensured through protection of data collected from the participants.

## Results

**Socio-demographic characteristics**: the mean age of the respondents was 35.7±9.5 years. Majority, (37.6%) were in the 30 to 39 years age group. Most (84.1%) of the respondents were females, 78.0% were married, and 63.0% were Christians. Almost all (96.6%) were of the Yoruba ethnic group. Forty-seven percent (46.5%) were community health extension workers and 41.3% had less than 5 year´s work experience. One hundred and fifty four (47.1%) of the primary health care workers were from the urban area (Table 1).

**Table 1:** socio-demographic characteristics of respondents (N = 327)

Variables	Frequency n (%)
**Age group (years)**	
< 20	9(2.8)
20 - 29	80(24.5)
30- 39	123(37.6)
40 - 49	77(23.5)
≥50	38(11.6)
**Sex**	
Male	52(15.9)
Female	275(84.1)
**Marital status**	
Single	70(21.4)
Married	255(78.0)
Widowed	2(0.6)
**Religion**	
Christianity	206(63.0)
Islam	121(37.0)
**Length of practice (years)**	
1 - 4	135(41.3)
5 - 9	66(20.2)
10 - 14	47(14.4)
15 - 19	38(11.6)
≥20	41(12.5)
**Monthly income (in naira)**	
< 50,000	134(41.0)
50 - 99,999	86(26.3)
≥ 100,000	74(22.6)
Not paid	11(3.4)
No response/missing value	22(6.7)
**Region**	
Urban	154(47.1)
Semi urban	173(52.9)
**Category of health workers**	
Doctor	10(3.1)
Nurse/midwife	36(11.0)
Lab attendant	39(11.9)
CHEW	152(46.5)
Medical record officer	46(14.1)
CHO	25(7.6)
Others	19(5.8)

Others = Voluntary health workers, Health Assistants, Pharmacist, Nutritionist and Health educators. CHEW = Community Health Extension Workers. CHO = Community Health Officer

**Awareness of neglected tropical diseases**: respondents had some level of awareness on NTDs namely; Schistosomiasis (78%), Rabies (64.5%), Onchocerciasis (57.2%), Trachoma (40.7%), Soil-transmitted helminths (37.3%), Buruli Ulcer (33.3%), Lymphatic filariasis (31.2%) and Human African Trypanosomiasis (31.2%). Others such as leprosy, dracunculiasis, dengue fever, chagas disease, yaws, leishmaniasis, mycetoma, chikungunya, and echinococcosis had 8.5% awareness collectively.

**Knowledge of neglected tropical diseases**: as shown in Table 2, 187 (57.2%) of the respondents had good knowledge of NTDs.

**Table 2:** distribution of knowledgeable respondents on neglected tropical diseases (N= 327)

Knowledge variables	Frequency n (%)
Causes of NTDs transmission*	
Exposure to excessive heat from the sun	117 (35.8)
Lack of access to safe water and sanitation	277 (84.7)
Live in an unclean environment	262 (80.1)
Overworking	153 (46.8)
Presence of abundant vectors	218 (66.7)
Signs and symptoms of NTDs*	
Rash/itching	274 (83.8)
Sore throat	81 (24.8)
Sweating	97 (29.7)
Blood in urine	226 (69.1)
Sleeplessness	193 (59.0)
Strategy for combating NTDs*	
Preventive chemoprophylaxis	225 (68.8)
Anti-retroviral therapy	108 (33.0)
Resting	111 (33.9)
Safe drinking water and sanitation	262 (80.1)
Good hygiene and education	269 (82.3)
Case management	229 (70.0)
Drug type for treatment of NTDs*	
Ivermectin	213 (65.1)
ACT	129 (39.4)
Praziquantel	182 (55.7)
Ibuprofen	131 (40.1)
Albendazole	193 (59.0)
Preventive chemotherapy NTD*	
Rabies	63 (19.3)
Schistosomiasis	170 (52.0)
Buruli ulcer	72 (22.0)
Onchocerciasis	143 (43.7)
Yaws	75 (22.9)
Neglected Tropical Diseases can be transmitted from one person to another	252 (77.1)
Neglected Tropical Diseases can be treated	310 (94.8)
Neglected Tropical Diseases a problem of public health importance in Nigeria	262 (80.1)
**Overall knowledge of NTDs**	187 (57.2)

* = multiple response; ACT = Artemisin Combination Therapy Mean = 16.2, Median = 17.0, Mode = 17.0, SD = 5.5

**Knowledge of risk factors for NTDs transmission**: majority, (84.7%) knew lack of access to clean water and sanitation was a risk factor. A large proportion (80.1%) stated that living in an unclean environment, while 66.7% stated presence of vectors. However, about one-third, (32.7%) had misconceptions that NTDs was transmitted through exposure to excessive heat from the sun, while 20.8% mentioned over-work.

**Knowledge of prevention and control strategies for NTDs:** a high proportion of the respondents, (82.3%) knew good hygiene, provision of safe drinking water (80.1%), and the use of preventive chemotherapy (68.8%) as ways to prevent or control NTDs. About one-third, (32.4%) and one-quarter (40.4%) of the respondents stated rest and use of anti-retroviral therapy respectively.

**Knowledge of signs and symptoms of NTDs**: most of the primary health care workers, (83.8%) had knowledge that rash/itching is associated with NTDs, 226 (69.1%) reported blood in urine, while 193 (59.0%) identified lack of sleep. Meanwhile, 151 (46.2%) answered “yes” to sore throat as a possible symptom of NTDs, while 130 (39.8) stated sweating.

Table 3 showed there was a statistically significant difference in the mean knowledge score between urban and semi-urban primary health care workers. Primary health care workers in the urban areas were more knowledgeable about NTDs (16.83±5.08) than their counterparts in the semi-urban areas of the state (15.64±5.86) [t=1.956; p=0.018]. Although, male primary health care workers (16.33± 6.17) had more knowledge on NTDs than their female counterpart (16.18±5.41), the difference was not strong enough to yield a statistical significant result [t= 0.182; p= 0.097]. A statistical difference in mean knowledge score was recorded for the monthly income of respondents [F= 7.443; P= <0.001]. Post-hoc test indicated mean score of health care workers who receive 100 thousand naira and above monthly (18.55±4.77) was significantly different from those that were earning less than 50 thousand naira monthly (15.09±5.39) and health care workers earning between 50 to 99,999 thousand naira monthly (15.85±6.04). Furthermore, statistical significant difference in mean knowledge score was recorded based on category of health worker [F= 3.703; P= 0.001]. Post-hoc test indicated mean score of nurses (18.42±4.13) was significantly different from those of CHEWs (15.39±5.53) and medical record officers (14.76±5.84).

**Table 3:** respondents mean NTDs knowledge score by demographic characteristics

Variables	N	Mean (SD)	Test values	p-value
**Sex**			0.182b	0.097
Male	52	16.33 (6.17)		
Female	275	16.18 (5.41)		
**Religion**			0.643b	0.157
Christianity	206	16.35 (5.31)		
Islam	121	15.94 (5.89)		
**Region**			1.956b	0.018
Urban	154	16.83 (5.08)		
Semi urban	173	15.64 (5.86)		
**Monthly income (N)**			7.443a	<0.001
> 50,000	134	15.09 (5.39)		
50,000 - 99,999	86	15.85 (6.04)		
≥100,000	74	18.55 (4.77)		
Not paid	11	18.73 (4.47)		
**Category of health workers**			3.703a	0.001
Doctor	10	20.00 (6.04)		
Nurse/midwife	36	18.42 (4.13)		
Lab attendant	39	16.90 (5.88)		
CHEW	152	15.39 (5.53)		
Medical record	46	14.76 (5.84)		
CHO	25	18.28 (4.24)		
Others	19	15.79 (5.27)		

a= F-values from one-way ANOVA; b= Independent t-test values

**Factors influencing recognition of neglected tropical diseases**: two hundred and sixty-four (80.7%) respondents reported they could recognize any case of an NTD when it presents. Previous training on NTDs was a significant predictor for recognizing these diseases, health care workers who had received training were 7 times more likely to recognize an NTD (AOR = 7.09, CI= 3.15 - 15.93) compared to those who had not received such training. Respondents with 15 - 19 years´ of work experience were more likely to recognize NTDs compared to respondents with less than 5 years´ work experience (AOR=4.65, CI= 1.20 - 18.09). Although age was significantly associated with NTDs recognition, the association was between those aged 30 - 39 when compared to their counterparts aged 50+ (AOR= 3.40, CI= 1.20 -9.63) (Table 4).

**Table 4:** factors associated with recognition of neglected tropical diseases

Variables	Can recognize an NTD (N=264)	Unadjusted	Adjusted	p-value
	**n (%)**	**OR (95% CI)**	**OR (95% CI)**	
**Age group (years)**				
<20	08 (3.0)	3.69(0.41- 32.94)		0.242
20 - 29	62 (23.5)	1.59(0.67- 3.77)		0.292
30 - 39	103 (39.0)	2.38(1.03- 5.48)	3.40(1.20- 9.63)	0.021*
40 - 49	65 (24.7)	2.50(1.00- 6.28)		0.051
≥ 50	26 (9.8)	1	1	
**Length of practice (years)**				
<5	102 (38.6)	1	1	0.319
5 - 9	54 (20.5)	1.46 (0.70- 3.05)		0.099
10 - 14	41 (15.5)	2.21 (0.86- 5.67)		0.027*
15 - 19	35 (13.3)	3.78(1.09- 13.08)	4.65(1.20- 18.09)	
≥ 20	32 (12.1)	1.15 (0.50- 2.66)		0.743
**Training on NTDs**				
Yes	128 (48.5)	6.47(2.97-14.12)	7.09(3.15- 15.93)	<0.001*
No	136 (51.5)	1	1	
**Knowledge of NTDs**				
Good	157 (59.5)	1.61(0.93-2.80)		0.089
Poor	107 (40.5)	1		
**Availability of tools for NTD diagnosis**				
Yes	61 (23.1)	2.07(0.93-4.57)		0.074
No	203 (76.9)	1		

* =p-value for Adjusted Odds Ratio; Significance level (p) <0.05

**Factors influencing the reporting of neglected tropical diseases**: out of 159 respondents that reported they had seen an NTD prior to this study, only 101 (63.5%) said they notified the disease surveillance and notification officer. Training on disease surveillance reporting was significantly associated with reporting of NTDs. Primary health care workers who had received training on disease surveillance reporting were 3 times more likely to report NTD (AOR= 3.41, CI=1.17 - 9.92) compared to those who had not received such training. Health care workers who had reporting forms in their facilities were 3 times more likely to report NTDs (AOR= 3.17, CI=1.07 - 9.35) than those who do not have the forms in their facility. Additionally, primary health workers who received feedback on a previously reported disease, were 4 times more likely to report NTDs than those who did not receive feedback (AOR= 4.12, CI=1.32 - 12.80) (Table 5).

**Table 5:** factors associated with reporting of neglected tropical diseases

Variables	Ever reported NTD (N=101)	Unadjusted	Adjusted OR (95% CI)	p-value
	**n (%)**	**OR (95% CI)**		
**Ever had training on disease Surveillance reporting**				
Yes	69 (68.3)	5.66 (2.78- 11.54)	3.41 (1.17- 9.92)	0.024*
No	32 (31.7)	1	1	
**Availability of reporting forms in facility**				
Yes	83(82.2)	8.12 (3.88- 17.01)	3.17 (1.07- 9.35)	0.037*
No	18 (17.8)	1	1	
**Ever read the guidelines for surveillance reporting**				
Yes	66(65.3)	8.06 (3.71- 17.47)	NS	<0.001
No	35 (34.7)	1		
**Ever been visited for supportive supervision**				
Yes	66(65.3)	3.27(1.67- 6.43)	NS	0.001
No	33(32.7)	1		
**Receive regular feedback on disease reported**				
Yes	63(62.4)	6.09 (2.90- 12.82)	4.12 (1.32- 12.80)	0.014*
No	35(34.7)	1	1	
**Ever received a penalty for not reporting diseases**				
Yes	38 (37.6)	4.60 (1.89- 11.20	NS	0.001
No	59 (58.4)	1		
**Feel reporting form is easy to understand**				
Yes	71 (70.3)	4.00(1.96 - 8.15)	NS	<0.001
No	25 (24.8)	1		
**Availability means to handle potential adverse effect in my facility**				
Yes	40 (39.6)	2.63(1.24- 5.60)	NS	0.012
No	57(56.4)	1		
**Require monetary incentive to report NTDs**				
Yes	44 (43.6)	3.06(1.44- 6.48)	NS	0.004
No	54 (53.5)	1		

* = p-value for Adjusted Odds Ratio; Significance level (p) <0.05; NS= Not significant for Adjusted Odds Ratio

**Reporting practice of respondents**: more than one-third, (48.6%) of the respondents reported they had identified at least a case of an NTD prior to this study. Some (22.3%) of the health workers identified schistosomiasis, followed by onchocerciasis (10.7%) rabies (7.0%), lymphatic Filariasis (6.1%) and soil-transmitted helminthes (3.4%). However, only 101 (63.5%) stated they notified the DSNO. Forty (39.6%) reported through phone call, 33 (32.7%) verbally reported to the head of their facility, while 28 (27.7%) reported through text messages.

**Reasons for not notifying neglected tropical diseases**: two hundred and twenty six respondents had never notified an NTD prior to this study. About two-third, (57.1%) claimed they did not know the reporting procedure, while 70 (31.0%) believed reporting will be done by someone else. Other reasons for not notifying include patient confidentiality (22.6%), not knowing the importance of reporting (11.1%) and too busy to report (8.4%).

## Discussion

Healthcare workers poor knowledge and skill in understanding, diagnosing and management of NTDs is one of the challenges to the control and elimination of these diseases. Assessing provider´s knowledge of NTDs and identifying factors that influences recognition and reporting, are vital for policy enactment and health care service provision. With limited vector control measures and poor environmental sanitation dominant in many communities in Nigeria, coupled with lack of vaccines for many of the NTDs, control of the disease in the country to a large extent depends on early detection and treatment of cases. Prompt reporting of infectious diseases is vital for early intervention and monitoring of trends at the local, state and national levels. Despite its importance, reporting is often not done as reported by Tan *et al*. [21]. The overall knowledge of NTDs by healthcare workers was just fair. This is a major concern as it suggests that diagnosis of many of these diseases will be missed and invariably its treatment and control. A similar report of inadequate knowledge among health personnel was reported in a study by Zenq *et al*. in China and Awosolu in Nigeria for some of the NTDs and attributed it to lack of adequate training [22,23]. Health workers good knowledge of the signs and symptoms including treatment and control of the diseases was also inadequate. This is similar to findings by Awosolu who reported that only 18.5% and 22.8% of health professionals had good knowledge of the signs and symptoms as well as treatment and control of an NTD respectively [23]. Respondents showed highest knowledge of schistosomiasis than any other NTDs, this may be because of its endemicity and prevalence in the country [5,14]. Health workers also recorded high knowledge of Onchocerciasis. Similar report was documented in Abuja, Nigeria by Olamiju *et al*. [24]. The high level of knowledge could be attributed to the prevalence of the disease and the duration of its control programs which have been in existence since 1991 in Nigeria [15,24]. However, respondents had low knowledge of Chagas disease, Dracunculiasis, and Leishmaniasis.

These findings were consistent with studies reported by Gboeloh and Elele in Port Harcourt and Olamiju *et al*. in Abuja, Nigeria which recorded low knowledge in Chagas diseases and Leishmaniasis [24,25]. The poor level of knowledge may be due to the low prevalence of these diseases in the country and some geographical variations [26]. Urban primary health care workers were more knowledgeable about NTDs than those in rural areas, this scenario is similar to the findings by Adeoye *et al*. in southwest Nigeria and Kamga *et al*. in Cameroon [26,27]. This may be because health care workers in urban areas tend to have better access to health information than their counterparts in the rural areas [28]. It could also be attributed to the imbalance in the distribution of health workers because skilled health workers prefer to settle in the urban areas for better pay [29,30]. The knowledge about NTDs was better among male health workers. Studies from Cameroon by Kamga *et al*. and study by Olamiju *et al*. in Nigeria also reported similar findings, with males having higher knowledge scores on NTD activities [24,26]. It is alarming to know that some of the health workers had misconception on transmission of NTDs such as exposure to excessive heat from the sun. This highlights the need for education and routine training of health workers on these diseases. A high proportion of the respondents reported they could recognize an NTD when they see one. Although, only a few of them reported they had ever identified an NTD. Barriers identified to recognition of some NTDs among healthcare workers documented in some studies were poor knowledge and awareness of NTDs, lack of training and limited access to appropriate diagnostic tools and methods for NTDs diagnosis [22,23, 31]. Accordingly, short length of practice as a health worker and previous training on NTDs influenced recognition of NTDs. Unfortunately, only few health workers had received such training. Therefore, it is very important to address these internal and health system barriers by initiating training programs on NTDs and providing diagnostic tools in health facility to improve recognition of NTDs in Nigeria and other low income countries.

Our study also revealed the serious inadequacies in healthcare workers NTDs reporting. Poor reporting may be attributed to the fact that more than half of the respondents claimed they had never diagnosed a case of an NTD before this study. A high proportion of the respondents stated that they do not know the reporting procedure and thought notification would be done by someone else. This implies that many cases of these diseases may have been unidentified leading to serious under-reporting and underestimation [12-14]. Training on disease surveillance reporting was found to significantly improve reporting NTDs. However, only few health workers in our study had received training on disease reporting. Similar findings was reported by Bawa *et al*. among health workers in Yobe Nigeria, but contrary to the study by Dairo *et al*. in south-west Nigeria, which reported that many respondents had been trained on surveillance reporting [32,33]. Furthermore, non-availability of reporting forms was found to be associated with non-reporting of NTDs. Similar findings was reported by several studies among health workers in developing countries which showed lack of reporting forms as a reason for not reporting notifiable diseases [32-35]. Lack of feedback was associated with non-reporting of NTDs. Only few health workers had received feedback on diseases reported. This was consistent with other studies in Nigeria [32,36]. This is a concern because lack of feedback could be a demotivating factor for health workers in reporting NTDs in that, they may not see its importance, thus leading to poor performance in the future. Our study was limited in that recognition of NTDs was self-reported hence subject to information bias. It being a cross-sectional survey, causation could not be established, only association. Despite this, findings from the study are very useful as it will help inform policy and decision making towards NTDs. It will also provide useful information to improve knowledge, recognition and reporting among health workers on these diseases.

## Conclusion

This study showed that knowledge, recognition and reporting of NTDs among primary healthcare workers in Ibadan, Nigeria was inadequate. There were misconceptions about these diseases and their treatment modalities, which could pose a major threat to the success of NTDs control programs. Periodic in-service training of health workers to improve NTDs knowledge, and reporting is imperative. Supportive supervision and monitoring of health workers activities, regular feedback on diseases reported and provision of logistics to enable health care workers report NTDs effectively are urgently required.

### What is known about this topic

- Nigeria carries the highest burden of NTDs in sub-Saharan Africa;
- Ignorance, under-reporting, and lack of the skill to identify and treat NTDs has allowed the persistence of these diseases in many communities.

### What this study adds

- This study revealed that primary healthcare workers only had a fair knowledge of neglected tropical diseases;
- The study highlighted the areas that needs to be targeted to improve recognition and reporting of these diseases;
- This study provides baseline information for in-depth studies in other regions of the country.
